# The use of autologous platelet-rich fibrin matrix combined with meniscal repair in the treatment of parameniscal cyst: clinical results and cyst recurrence after 2-year of follow up

**DOI:** 10.1186/s40634-021-00423-1

**Published:** 2021-11-30

**Authors:** Daniele Screpis, Gianluca Piovan, Simone Natali, Stefano Pasqualotto, Stefano Magnanelli, Venanzio Iacono, Luca Farinelli, Marco Grassi, Claudio Zorzi

**Affiliations:** 1 Department of Orthopaedics IRCCS Ospedale Sacro Cuore Don Calabria, Negrar di Valpolicella, Verona, Italy; 2 Clinical Ortopaedics, Department of Clinical and Molecular Sciences, Università Politecnica delle Marche, Ancona, Italy

**Keywords:** Parameniscal cyst, PRP membrane, All-inside suture, PRP augmentation

## Abstract

**Purpose:**

Parameniscal cysts are associate with horizontal meniscal tears. Arthroscopic meniscal repair and the excision of the cyst by mini-open approach represent a valid treatment. However, the recurrence of cyst is still a current issue. Therefore, biological factors may be considered to promote the biological repair and avoid recurrence. The aim of the present study was to report the clinical results and the rate of recurrence of the cyst after minimum 2-year of follow up in a cohort of patients treated by meniscal repair and autologous platelet-rich fibrin matrix augment.

**Methods:**

Patients with lateral parameniscal cyst undergoing arthroscopic meniscal repair and autologous platelet-rich fibrin matrix augment between 2016 and 2019 were retrospectively reviewed in March 2021. Inclusion criteria were absence of prior surgery on the affected knee with minimum 2-year of follow-up. Exclusion criteria were concomitant ligament lesions, rheumatic diseases and knee osteoarthritis. After reviewing the database, each selected patient was contacted and asked to participate in the study; at the follow-up evaluation all patient signed an informed consent. Tegner-Lysholm knee score, IKDC and NRS were collected before surgery and at follow-up.

**Results:**

This study included 15 patients (8 male) with mean age of 32.8 years old. No recurrence of the cysts was observed. The Tegner-Lysholm knee score and IKDC subjective scores increased respectively from 41.3 ± 5.4 and 37.6 ± 5.1 at baseline to 92.3 ± 4.6 and 89.4 ± 2.6 at the final follow up. Concerning pain relief, the Numeric Pain Rating Scale (NRS) displayed a significant improvement reaching at the follow up a score of 1,3 ± 1.1 in comparison to 6.8 ± 0.9 at the baseline.

**Conclusion:**

Surgical management of symptomatic lateral parameniscal cyst with cyst excision, autologous PRP membrane application and meniscus repair demonstrated excellent subjective clinical outcome with any cyst reoccurrence.

**Level of evidence:**

III, retrospective cohort study.

## Introduction

Parameniscal cysts are defined as internal disorder of the knee joint located on the medial or on the lateral side that might be asymptomatic or cause knee pain, joint swelling and soft tissue masses [1]. Over the past years, most of the orthopedic literature focused on the cysts located on the lateral side because they were usually symptomatic [2, 3]. Despite the etiology is still doubtful, it is known that parameniscal cysts are associate with horizontal meniscal tears. It was hypothesized that the formation of a one-way valve mechanism might allow the accumulation of synovial fluid within a meniscal cavity, especially during weight-bearing and knee range of motion. In symptomatic patients the surgical treatment is mandatory. Nowadays, two arthroscopic techniques have been widely used for the treatment of parameniscal cysts: arthroscopic and mini-open excision which aim to remove the entire cyst by a mini-open approach after arthroscopic meniscal repair, and an entirely arthroscopic procedures consisted by meniscectomy and cyst decompression [4]. Unfortunately, in case of large cyst, subtotal meniscectomies might be necessary to carry out the decompression [5], therefore the combined arthroscopic and mini-open approach may be preferred especially in young patients.

It has been well established that cyst dimension, failure to disrupt the check-valve mechanism and multilobulated structure are associated with a cyst recurrence rate that could reach 15% of cases [6].

Due to the deep extend of the cyst in the red-red zone of the meniscus, biological factors may be considered to promote the biological repair. Common methods to enhance the vascularity and healing of the meniscus include trephination, synovial abrasion, application of a fibrin clot and platelet-rich plasma (PRP). PRP is characterized by a concentration of platelets above the baseline values [7]. It is theorized that higher levels of platelets allow the release of growth factors, which may promote angiogenesis and soft tissue healing [8, 9].It has been widely used in various orthopedic issues; particularly, recent papers reported promising and satisfying results following meniscal tissue repair with PRP augmentation [6, 10–14].

The purpose of this study was to evaluate the clinical results and the rate of recurrence of the parameniscal cyst in a cohort of patients treated by meniscal repair and autologous platelet-rich fibrin matrix augment. We hypothesized that the biological augmentation in meniscal healing could avoid cyst recurrence with satisfying clinical results.

## Materials and methods

### Patients

The collected data of patients who underwent implantation of autologous platelet-rich fibrin matrix for parameniscal cysts between 2016 and 2019 were retrospectively reviewed in March 2021. Inclusion and exclusion criteria were summarized in Table 1.

**Table 1:** Inclusion and Exclusion criteria

*Inclusion criteria*	*Exclusion criteria*
Lateral parameniscal cysts unresponsive to medical treatment^a^ for a minimum of 6 months	Anterior and/or posterior cruciate ligament tears
History of knee trauma	Concomitant collateral ligament tears
Knee MRI and X rays available before surgery	Previous ipsilateral femur or tibia fracture
Meniscal suture	Rheumatic diseases and osteoarthritis
No problem referred at knee before trauma	Partial meniscectomy
At least 24 months of follow up	Loss of follow up

^a^Rest, nonsteroidal anti-inflammatory drugs (NSAIDs) pain killers, physiotherapy, steroid and/or viscosupplementation injection

As per institutional policy, each patient undergoing this procedure was screened and documented by recording history and demographic data at the time of surgery. All patients underwent to knee X-Ray and Magnetic Resonance Imaging (MRI) as standard protocol before surgery. Moreover, the following clinical scores were evaluated: IKDC subjective, Numeric Pain Rating Scale (NRS) and Tegner-Lysholm knee score for the pre-injury level and pre-operative physical activity. After reviewing the database, each selected patient was contacted and asked to participate in the study; at the follow-up evaluation all patient signed an informed consent and underwent to physical examination. The same scores applied for the basal evaluation were used and recorded at follow-up.

Each patient that accepted to participate at the study, made up a new knee MRI at follow up, the images were collected. All procedures were conducted in accordance with good clinical practice and the Declaration of Helsinki 1964. The present study was approved by the Ethics Committee of Verona and Rovigo – Italy (protocol n. 61,386–19/09/2018).

### Surgical technique

**Fig. 1:**
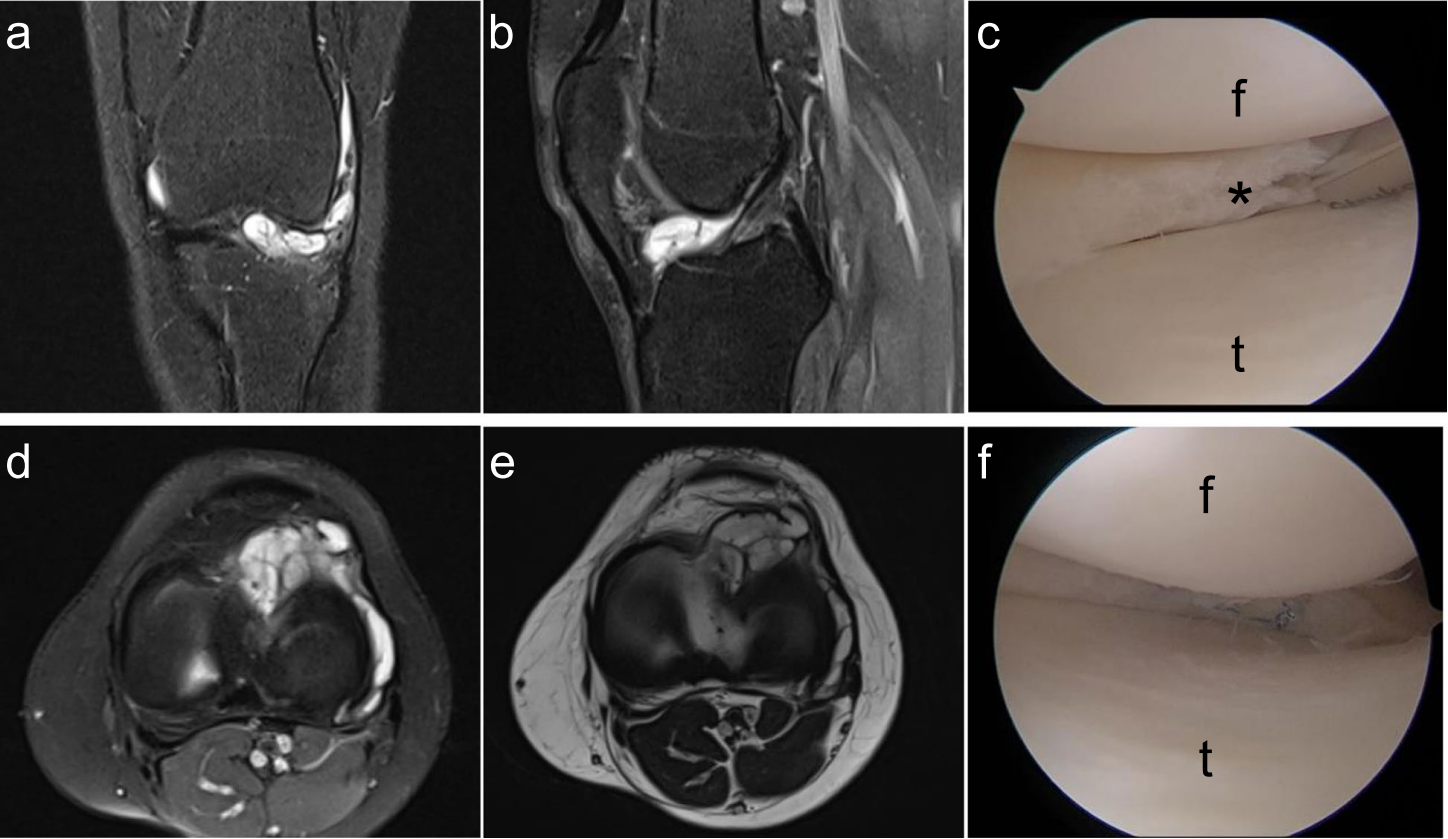
**a** and **b** Coronal and sagittal magnetic resonance image (MRI) of left knee with lateral-sided parameniscal cyst. **c** arthroscopic view of horizontal meniscal tear (*). **d** and **e** Axial view of MRI of the lateral-sided parameniscal cyst. **f** all-inside meniscal sutures. t: tibia, f: femur

General knee arthroscopic examination is routinely carried out via anterolateral and anteromedial portals. A diagnostic arthroscopy is conducted to ensure that there is no additional intra-articular pathology. Meniscal tears were treated by all-inside or outside-in suture (Fig. 1).

**Fig. 2:**
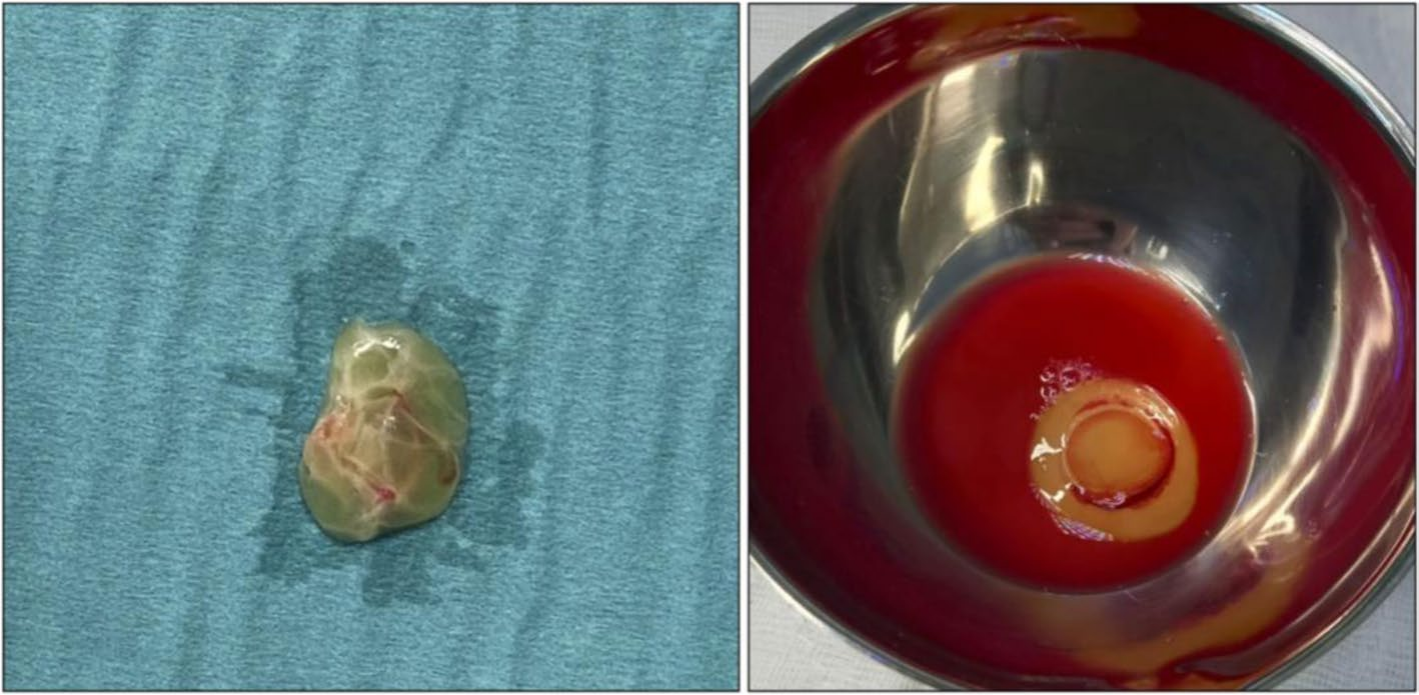
The autologous platelet-rich fibrin matrix membrane before implantation. The kit provides the membrane set within the platelet rich plasma of the patients (right figure)

The autologous platelet-rich fibrin matrix (RegenKit Extracell Membrane, Regen LAB, Switzerland) is a thin layer of autologous fibrine very rich in platelets. It is obtained from a blood sample of the patient following manufacturers’ instruction (Fig. 2).

**Fig. 3:**
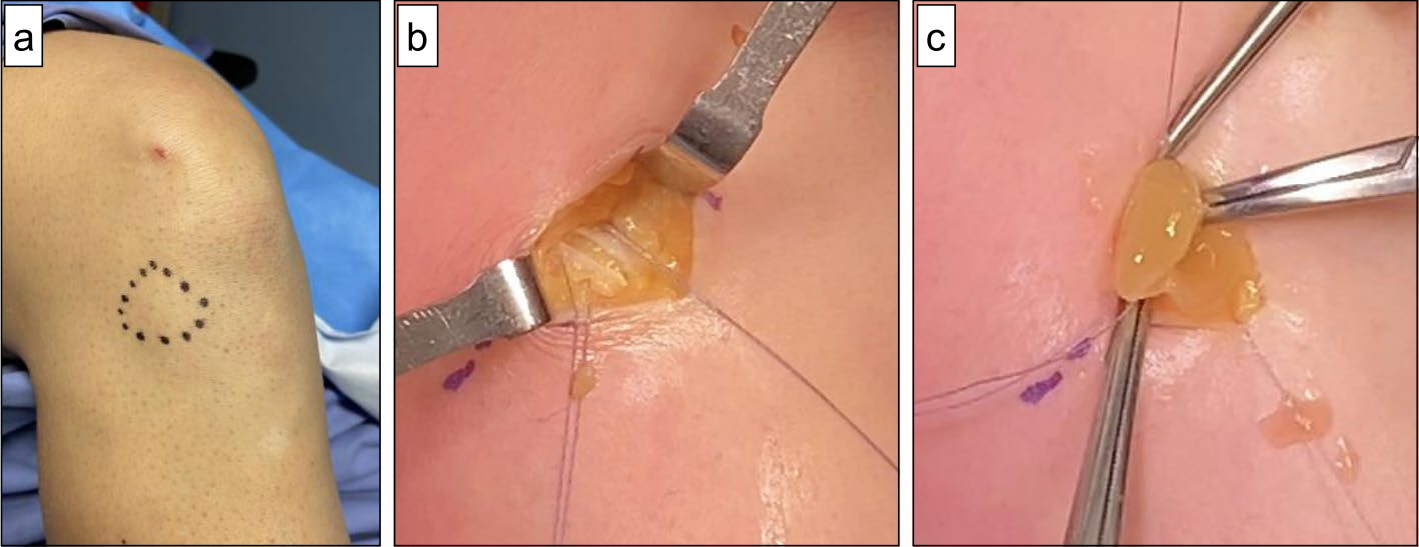
The application of autologous membrane. **a** Parameniscal cyst is marked with a sterile skin-marker. **b** A Z-shaped shaped incision is performed on the iliotibial band. Subsequently, a horizontal-oblique incision of the capsule is performed over the joint line in correspondence of the cyst. The cyst is evacuated **c**) The autologous platelet-rich fibrin matrix is sutured side-to-side to the adjacent capsule with an absorbable suture

A small horizontal or oblique skin incision is made over the joint line in correspondence of the cyst, and the subcutaneous tissue of the knee is dissected. Transillumination of the lateral compartment could determine the proper level for incision placement, especially when one is trying to make a smaller incision. Z-shaped incision is made at ileotibial band to improve exposure of underlying layers and closure. A horizontal-oblique incision of the capsule is performed over the joint line in correspondence of the cyst. The cyst is evacuated. The autologous platelet-rich fibrin matrix membrane is located in the outer layer of the lateral meniscus and then sutured side-to-side to the adjacent capsule by adsorbable suture [15] (Fig. 3).

### Post-operative protocol

Postoperatively, all patients start range of motion exercises immediately after the surgery. Physical therapy emphasizes early quadriceps muscle activation and knee flexion from 0° to 90° restricted for the first 4 weeks and progressed thereafter. Weight-bearing was allowed after 4 weeks.

### Statistical analysis

Continuous variables were assessed using the paired-sample Student’s t-test. Statistical significance was set at *p* value < 0.05. For data analysis, a dedicated statistical software was used (SPSS vs 23.0, Chicago, Illinois, USA).

## Results

**Fig. 4:**
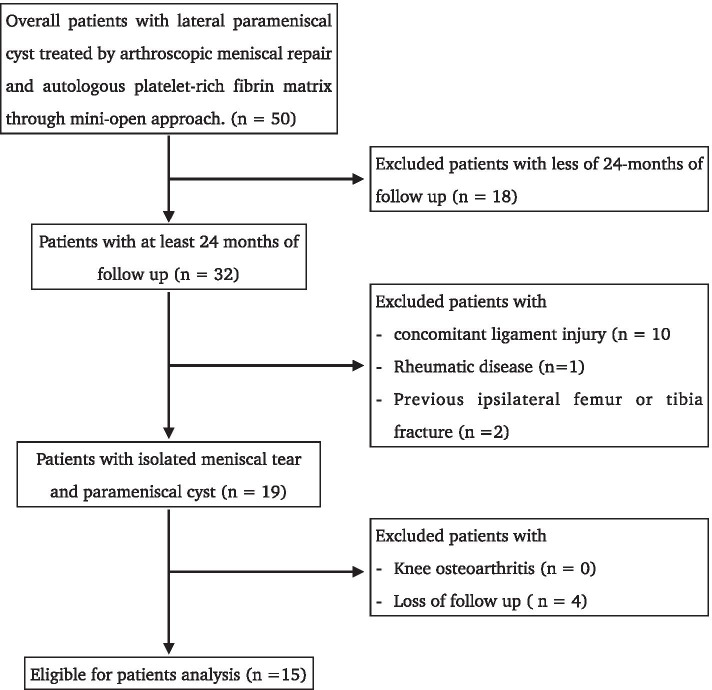
Eligible patients for analysis

Figure 4 showed the selection of eligible patients for analysis. After application of inclusion and exclusion criteria there were 15 patients eligible for the analysis. All patients were operated by the same surgeon. Table 2 showed the baseline characteristics of patients included in the study who underwent arthroscopic meniscal repair and application of autologous platelet-rich fibrin matrix augment. It is worth mentioning that most of patients were characterized by horizontal meniscal lesions (60%) located in body-posterior horn of lateral meniscus (93%).

**Table 2:** characteristics of patients included in the study. SD: standard deviation

Number of patients		15
Follow up, months: mean (range)		42.6 (33–58)
Male/Female, N. (%)		8 (53%)/7(47%)
Age, mean (range)		32,8 (21-53y)
Right/Left Knee: number (%)		10 (67%)/5 (33%)
Location of the meniscal tear		
	*Anterior horn, number (%)*	1 (7%)
	*body-posterior horn, number (%)*	14 (93%)
Pattern of meniscal tear		
	*Horizontal, number (%)*	9 (60%)
	*Complex, number (%)*	4 (26%)
	*Radial, number (%)*	2 (13%)
Cyst size, mean (SD)		2,9 ± 0,8 cm^3^

**Fig. 5:**
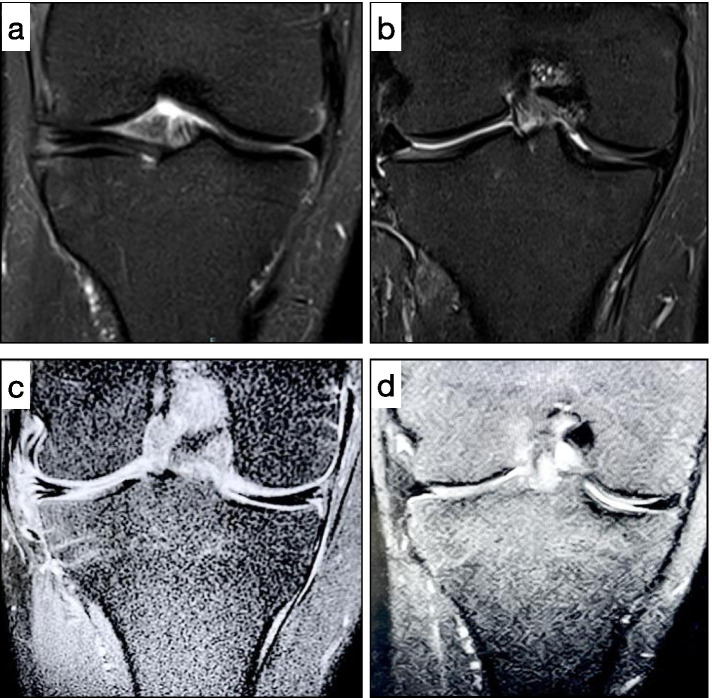
**a** Coronal magnetic resonance of horizontal lesion of patient with lateral parameniscal cyst, **b**) coronal magnetic resonance of the patient of a) after 36-month from surgery (partial meniscectomy and application of autologous PRP membrane; **c**) and **d**) coronal magnetic resonance of the same patient respectively at time of surgery and after 48 months from surgery. In both patient the meniscus appears healed with no recurrence of cyst

Fourteen patients underwent to meniscal suture through all inside system (Fast fix meniscal repair, Smith & Nephew, Andover, MA). One patient with meniscal tear located at anterior horn of lateral meniscus underwent to meniscal suture by outside-in technique. No cases of cyst recurrence were reported by clinical examination and follow-up MRI images (Fig. 5). Moreover, from follow-up MRI images we reported that the signal intensity (at fat suppressed T2-weighted) of previous meniscal tear was lower compared to the same tears before surgery in all patients.

No complications as infection or adverse effect of autologous membrane implantation have been reported during follow up period.

**Fig. 6:**
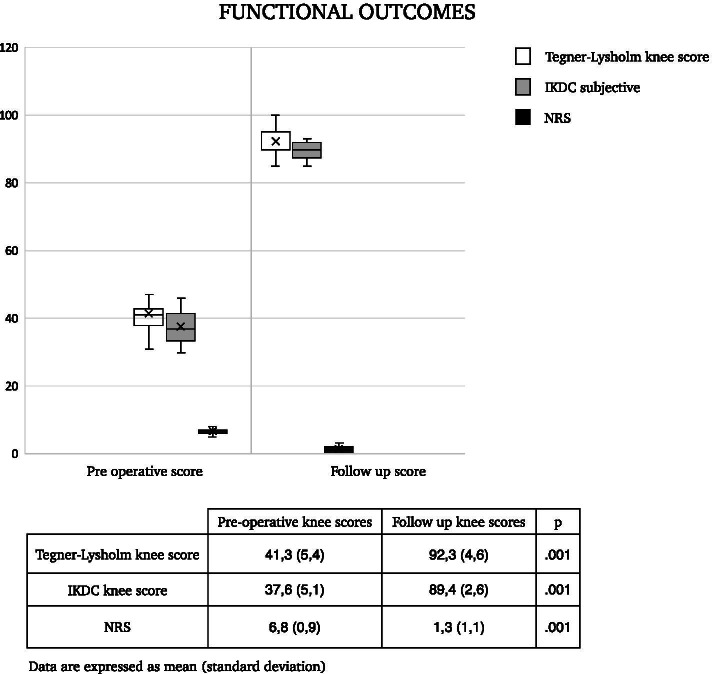
Functional outcomes

At a mean follow up of 42,6 months patients show a significant and clinical improvement in terms of pain relief and functional outcomes. The Tegner-Lysholm knee score increased from 41.3 ± 5.4 at the baseline to 92.3 ± 4.6 at the final follow up (*p* < 0.01). Likewise, IKDC subjective scores increased from 37.6 ± 5.1 at the baseline to 89.4 ± 2.6 at the follow-up (*p* < 0.01). Concerning pain relief, the Numeric Pain Rating Scale (NRS) displayed a significant improvement reaching at the follow up a score of 1,3 ± 1.1 in comparison to 6.8 ± 0.9 at the baseline (*p* < 0.01) (Fig. 6).

## Discussion

The most important finding of the present study was that the application of autologous PRP membrane for the treatment of parameniscal cyst represent a safe and efficacy method for augmentation of meniscal healing. No cases of infection or adverse effect of membrane implantation were reported during follow up period. Moreover, despite the considerable size of the cysts of our series, no cases of recurrence have been observed. Even though, previous studies reported that overall recurrence of parameniscal cyst ranged from 9.4 to 15% after arthroscopic decompression [16–18]. Each patient was clinical satisfied, and we reported a significant improvement of clinical results from baseline. Moreover, none underwent to following conservative or surgical knee treatment after the procedure during follow up period.

It is known that various cytokines from platelets have a myriad effect on cellular processes involved in tissue healing as cell proliferation, angiogenesis, cell chemotaxis, and matrix synthesis [19–21]. The autologous PRP membrane contains a high concentration of platelets that release GFs with healing properties. It is presumable that these GFs might favor meniscal healing as demonstrated by follow-up MRI images. Indeed, several reviews and meta-analysis pointed out that PRP augmentation in meniscal repair could effectively enhance the efficacy of arthroscopic repair of meniscal injury, reduce the failure rate and improve the clinical outcomes [22–25].

The membrane is easily suturable and it has been successfully used for the therapy of vascular ulcers and promising results have been reported in the treatment of rotator cuff tears [1, 26]. In the present research, the platelet-rich fibrin matrix has been used as a sort of autologous “patch” in order to reinforce the meniscocapsular junction in correspondence of the parameniscal cyst by a side-to-side suture in the inner layer of the capsule.

The limitations of our study included the small cohort, its retrospective and nonrandomized design, the short follow-up and lack of control group which may have led to bias. On the other hand, the post-operative MRI images could accurately assess the meniscal healing and recurrence.

## Conclusion

The surgical management of symptomatic lateral parameniscal cyst with meniscus repair, cyst excision and autologous PRP membrane application demonstrated excellent subjective clinical outcome with any cyst reoccurrence at minimum 2 year of follow up.
